# Soil Enzymes and Antioxidants Activities of Edible Vegetables Grown in Soils Polluted by Gas Flaring

**DOI:** 10.21315/tlsr2023.34.2.2

**Published:** 2023-07-21

**Authors:** Doris Akachukwu, Paul Ndubuisi Anyiam, Polycarp Nnacheta Okafor, Chiedozie Ibegbulem, Ifeoma Irene Ijeh

**Affiliations:** 1 Department of Biochemistry, College of Natural Science, Michael Okpara University of Agriculture, Umudike, P.M.B 7267 Umuahia, Abia State, Nigeria; 2 Department of Biochemistry, School of Biological Sciences, Federal University of Technology, Owerri, Nigeria

**Keywords:** Antioxidant Enzymes, Gas Flaring, Plants, Pollution, Soil Enzymes

## Abstract

Associated gas flaring has several consequences on the environment. This study was aimed at assessing the impact of gas flaring on soil enzymes and plant antioxidant activities from gas flare-bearing communities in Nigeria. Soil and plant samples were obtained from farmlands in Ukwa West and Izombe gas flaring sites, as well as unpolluted site from Olokoro (used as control). The level of activities of soil urease, dehydrogenase, phosphatases, plant antioxidant enzymes and lactate dehydrogenase (LDH) of selected plants (*Gnetum africanum* [GA], *Piper guineense* [PG], *Gongronema latifolium* [GL], *Pterocarpus mildbraedii* [PM]) were evaluated using standard methods. The results showed that the activities of urease were significantly higher (*P* < 0.05) in soil from Ukwa site than Izombe and the control soil. Dehydrogenase (DHA) and phosphatases recorded higher activities (*P* < 0.05) for Izombe soil than in Ukwa compared with the control. For plants, superoxide dismutase (SOD), glutathione S-transferase (GST) and glutathione peroxidase (GPx) recorded a significant (*P* < 0.05) higher activities in all the plants assayed from Ukwa site than Izombe and the control site. The activities of GPx from GA and PG plants at Izombe site were not significant (*P* > 0.05) when compared with the control, except for PM and GL which recorded a significant decrease (*P* < 0.05) in GPX and SOD activities, respectively. The activities of catalase enzyme also decreased significantly (*P* < 0.05) in all plants grown at Ukwa, while an increase was seen for GA and PM grown at Izombe compared with control. The overall variability in enzymes activities is an indication that soil ecosystem and plants are altered significantly by the stress load from the gas flaring pollutants which could serve as bio-indicators for assessing ecological risks and bioremediation.

**Highlights**

- We investigated the effects of gas flaring on activities of soil enzymes and plant antioxidants grown in soils polluted by gas flaring.
- Gas flaring significantly increased the activities of soil enzymes and induced variations in plant antioxidants activities.
- These soil enzymes and plant antioxidants could serve as early bio-indicators of ecological risk and monitoring bioremediation.

## INTRODUCTION

Nigeria is one of the main producers of oil in the world. Oil exploration in the country has resulted in higher rate of gas flaring, which is the decomposition of associated gas from crude oil by burning (Abua & Ashua 2015). This practice sends poisonous pollutants directly into the sorrounding environment causing serious environmental and public health concern within nearby communities (Ajugwo 2013; Anacletus *et al*. 2014). While this practice of burning off associated gas from crude oil has been reduced to minimum in Western world, Nigeria still flares a significant amount of this gas into the environment which has grown proportionately with oil production over the years (Anacletus *et al*. 2014; Adewale & Mustapha 2015). Data from World Bank (2004) showed that Nigeria is the first among first 10 countries responsible for 75% of gas flaring pollutants in the world. This gas when properly managed has enormous advantage in generating various forms of energy including electricity and cooking gas. The constituents of flared gas are the most persistent organic pollutants in the environment (World Bank 2004; Abua & Ashua 2015). The low rate of their decomposition triggers their accumulation in soil and plants tissues thereby affecting soil quality which is mainly governed by transformation of inorganic compounds by enzymes from microorganisms. These enzymes include the representative of every enzyme class such as oxidoreductases, hydrolases, isomerases, ligases, liases and transferases (Garcia-Gil *et al*. 2013; Adetunji *et al*. 2017) which catalyse almost all transformation reactions in the soil including the conversion of organic substances and energy (Gu *et al*. 2009).

High temperatures and poisonous pollutants emanating from gas flaring have the tendency of impacting soil enzymes negatively and induce stress in plants which ultimately leads to a decrease in plant growth and productivity. In plants, abiotic stresses are inevitably linked with the elevated level of reactive oxygen species (ROS) (Jain *et al*. 2020) which induces cellular damage by degrading important plant proteins, inhibiting essential enzymes, altering gene expressions and interfering in various metabolic reaction pathways (Choudhury *et al*. 2013). Plants, as general strategy for adaptation, use the activation of various antioxidant enzymes to arrest the oxidative stress (Mittler 2002; Jain *et al*. 2020). However, oxidative damages can still occur as a result of imbalance between the production of ROS and antioxidant defense capacity of plant which triggers a chain reaction in plant cells leading to cell damage (Ahmad *et al*. 2010; Sharma *et al*. 2012). By arresting the stressors, the antioxidant enzymes in plants plays a crucial role in controlling the lifetime of generated ROS signals and preventing uncontrolled oxidation due to environmental stress and pollutants.

*Gnetum africanum* (ukazi), *Piper guineese* (uziza), *Gongronema latifolium* (utasi) and *Pterocarpus mildbraedii* (oha) are important local green vegetables and spices used in preparing soup in many homes in South-East Nigeria. They also possess medicinal property and have been widely used traditionally in treatment of diseases. Previous studies have reported the negative effects of gas flaring on phytochemicals in vegetable plants (Anacletus *et al*. 2014), crop nutrients (Akachukwu *et al*. 2018), vegetation (Seiyaboh & Izah 2017), water and air quality (Ekanem 2001), man’s health (Oniero & Abroibo 2001) and economy (Oghifo 2001) including its contribution to changing climate (Okeagu *et al*. 2006), environment (Achi 2003) and poverty among rural dwellers in Nigeria (Gabriel 2004). However, limited information is available in the literature to support the biochemical processes behind the harmful effect of flaring on soil and plants.

Therefore this study was designed for the purpose of evaluating the effect of flaring gas on soil and vegetable plants by looking at the enzymatic activities in response to the pollutant-induced stress.

## MATERIALS AND METHODS

### Study Area

**Figure 1:**
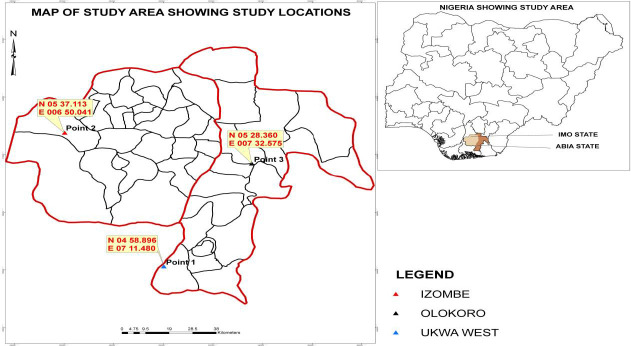
Study area map showing the locations of the study sites in Nigeria.

Ukwa (N 04°58.896, E 07°11.480) is located in Ukwa-West Local Government Areas (LGA) of Abia State while Izombe (N 05°37.113, E 006°50.041) is located in Oguta LGA of Imo State (Fig. 1). Both areas are rich in crude oil reserves and are locations to gas flaring points in South-East Nigeria. The people of Ukwa and Izombe are mainly traditional farmers who largely depend on crops they cultivate as source of livelihood. The cultivated farms in these regions are in close proximity with point of flare and to people’s homes. Therefore, it is in this context that this study on the impacts of the pollutants from gas flaring on soil and plant enzymes becomes necessary.

### Collection of Samples

Informed consent was obtained from local farmers prior to the collection of soil and plant samples from each region of the gas flare farmlands. Soils were obtained from a depth of 20 cm (3 km) with the aid of a clean cutlass. The sample used as control was collected from a non-gas flaring site in Olokoro, Umuahia South L.G.A, Abia State (N 05°28.360, E 007°30.575). Larger soil particles and aggregates were removed with the aid of a spatula. Each soil sample was properly mixed and kept in dark coloured polythene bags that were stored in ice-packed cooler to avoid loss of moisture and enzymatic reactions. Matured edible leaves of GA, PG, GL and PM were harvested in the morning hours from parent stock and authenticated at the Taxonomic Unit Department of Plant Science and Biotechnology, Michael Okpara University of Agriculture, Umudike with voucher specimen numbers of MOH 0126, MOH 0124, MOH 0125 and MOH 0127, respectively. The plant samples were wrapped in clean black polythene bags and stored in an ice-packed container to avoid enzymatic reactions. All the collected samples were moved to the laboratory same day for biochemical assay.

### Enzyme Extraction Procedure

The extraction of the enzymes from the samples was achieved following the methods of Tabatabai and Fu (1992). Twenty grams (20 g) of each soil and plant sample was grinded separately in 50 mL phosphate buffer. The extract was properly centrifuged at temperature of 4°C and speed of 15,000 rpm for period of 20 min. The supernatant was collected and used for the enzyme assay in each sample.

### Soil Urease Activity

The activity of soil urease (EC.3.5.1.5) was evaluated using the description of Hoffmann and Teicher (1961). Procedure: Toluene (0.25 mL), citrate buffer (pH 6.7) and 1 mL of urea substrate solution were added to a tube containing about 1 g of soil sample. The resulting solution was incubated at 37°C. The formation of ammonium after incubation was determined spectrophotometrically (UV-VIS, LABMAN) at a wavelength of 578 nm. Result was expressed as μgNg^−1^ dry soil.

### Evaluation of Dehydrogenase (DHA) Activity

Dehydrogenase activity was assayed by the reduction of 2,3,5-triphenyltetrazolium chloride (TTC) following the methods described by Casida *et al*. (1964). Procedure: 6 g of soil sample from each flaring region was thoroughly mixed with 120 mg CaCO_3_. 1 mL of 3% (W/v) TTC and distilled water (5 mL) was added and incubated at 30°C for 20 h for red colour development. This was followed by extraction with ethanol, filtered and absorbance taken at 485 mm. The concentration of DHA was expressed in μg TPF g^−1^min^−2^.

### Assessment of Acid and Alkaline Phosphatase Activity

Both enzymes were assayed from soil samples following the methods described by Tabatabai and Bremner (1969). Procedure: 4 mL of universal buffer solution was added into 1 g of soil sample in a flask for pH adjustment to 6.5. A quantity of 0.25 mL of toluene and 1 mL of 0.15 aluminum disodium p-nitrophenyl phosphate tetrahydrate were also added to the mixture followed by incubation at 37°C using water bath for an hour. Subsequently, 1 mL CaCl (0.5M) and 4 mL of aluminum sodium hydroxide (0.5M) were added to each flask and thoroughly mixed. The resulting solution was filtered, and absorbance measured at 420 nm.

### Plant Antioxidant System Assay

The activity of superoxide dismutase (SOD) from each plant collected from the flaring site was assayed following the description of Das *et al*. (2000). Catalase activity was measured following the methods of Sinha (1972) using hydrogen peroxide as substrate. Glutathione-S-transferase (GST) activity in each plant was carried out by the method of Habig *et al*. (1974), Glutathione peroxidase was according to the method of Rotruck *et al*. (1973) with hydrogen peroxide in the presence of GSH while lactate dehydrogenase (LDH) activity was assayed following the methods described by Weisshaar *et al*. (1975). The reduction rate of NAD is directly proportional to the LDH activity in the sample.

## DATA ANALYSIS

SPSS analytical package (version 20.0) was used for the analysis of the data generated. The differences between means of variables were compared with ANOVA and the least significant difference (LSD) was used to assess the least significant difference at *P* < 0.05 and results presented as graph of mean ± standard deviation of triplicate determinations.

## RESULTS

**Figure 2:**
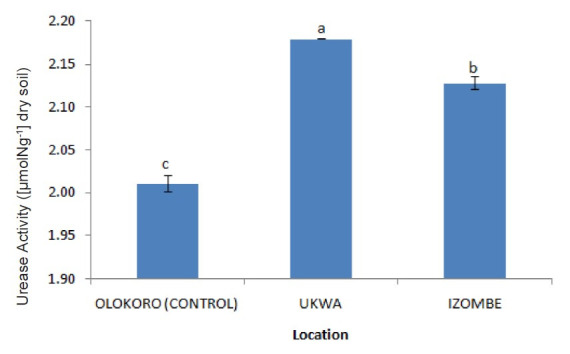
Effect of gas flaring on urease activity in soils from test site.

**Figure 3:**
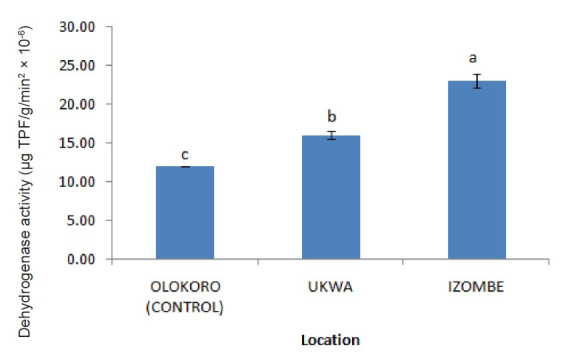
Effect of gas flaring on DHA activity in soils from test sites.

**Figure 4:**
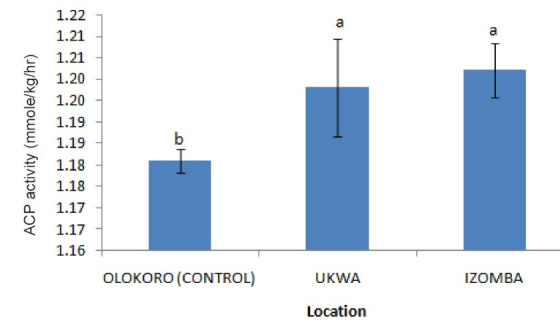
Effect of gas flaring on ACP activity in soils from test sites.

**Figure 5:**
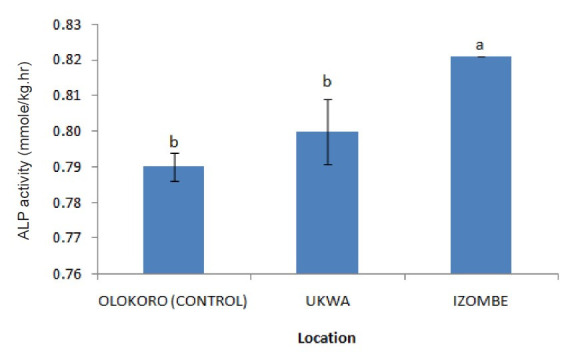
Effect of gas flaring on ALP activity in soils from test sites.

**Figure 6:**
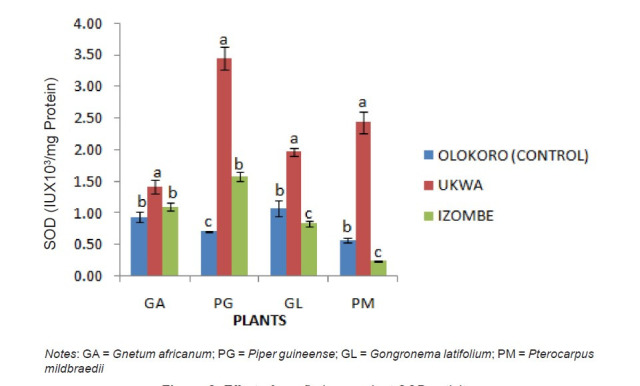
Effect of gas flaring on plant SOD activity. *Notes*: GA = *Gnetum africanum*; PG = *Piper guineense*; GL = *Gongronema latifolium*; PM = *Pterocarpus mildbraedii*

**Figure 7:**
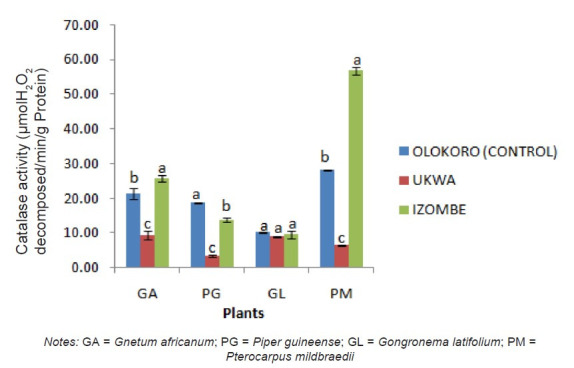
Effect of gas flaring on catalase activity of plant leaves from test sites. *Notes:* GA = *Gnetum africanum*; PG = *Piper guineense*; GL = *Gongronema latifolium*; PM = *Pterocarpus mildbraedii*

**Figure 8:**
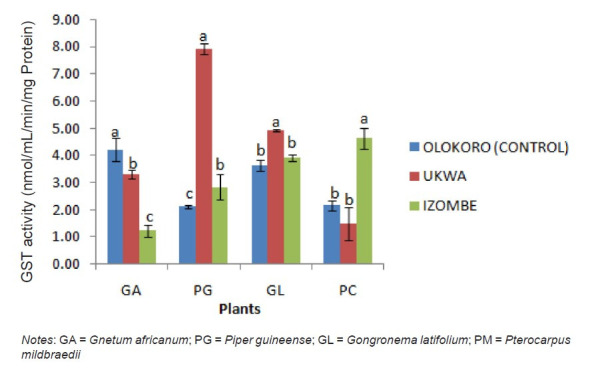
Effect of gas flaring on GST activity from plant leaves of test sites. *Notes*: GA = *Gnetum africanum*; PG = *Piper guineense*; GL = *Gongronema latifolium*; PM = *Pterocarpus mildbraedii*

**Figure 9:**
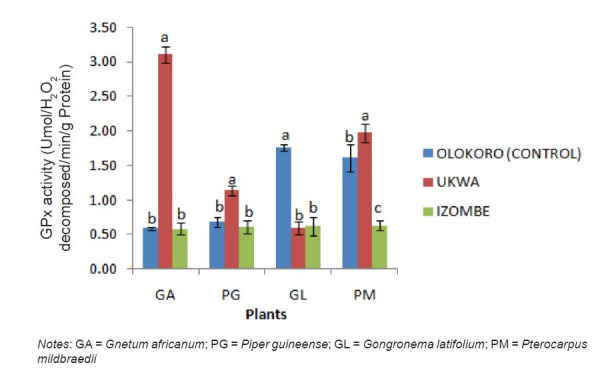
Effect of gas flaring on GPx activity from plant leaves of test sites. *Notes*: GA = *Gnetum africanum*; PG = *Piper guineense*; GL = *Gongronema latifolium*; PM = *Pterocarpus mildbraedii*

**Figure 10:**
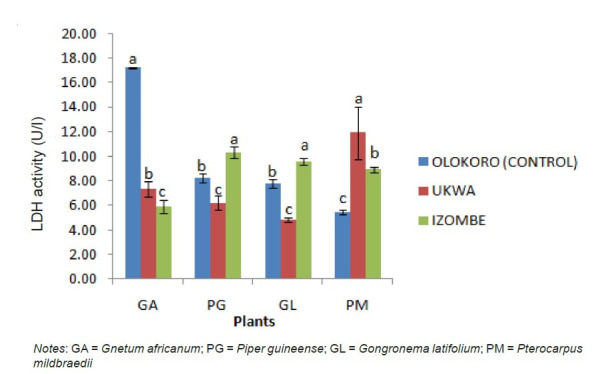
Effect of gas flaring on LDH activity from plant leaves of test sites. *Notes*: GA = *Gnetum africanum*; PG = *Piper guineense*; GL = *Gongronema latifolium*; PM = *Pterocarpus mildbraedii*

The effect of gas flaring on soil urease activity from the test locations (Ukwa and Izombe) is presented on Fig. 2. Urease from soil at Ukwa site recorded a higher activity (*P* < 0.05) than Izombe and the control soil. The activity of DHA increased significantly (*P* < 0.05) in soils collected from Izombe that Ukwa and control site which recorded the least activity (Fig. 3). Results of the effect of gas flaring on acid phosphatase (ACP) activity in soils from test sites (Ukwa and Izombe) as well as in the control site (Olokoro) are as presented on Fig. 4. The data showed an increased in ACP activity (*P* < 0.05) from Ukwa and Izombe soil respectively, compared with the control soil. The difference between ACP activity in Ukwa and Izombe site was not significant statistically (*P* > 0.05). Fig. 5 shows the impact of gas flaring on alkaline phosphatase activity in soils from test sites. A significant increase (*P* < 0.05) in soil alkaline phosphatase (ALP) activity from Izombe site was observed when compared with the control soil from Olokoro community. Again, no difference (*P* > 0.05) was seen between ALP activity from Ukwa and control soil. The results of the effect of gas flaring on SOD activity from plant leaves of test sites (Ukwa and Izombe), are presented in Fig. 6. The finding revealed that the SOD activity of all the plant samples from Ukwa site was higher (*P* < 0.05) compared with Izombe and control. All the plants collected from Ukwa site had increased catalase activity compared to that of Izombe and control (Fig. 7) except for *G. latifolium* which showed no significant effect. The effect of gas flaring on GST is represented in Fig. 8. The GST activity of *G. africanum* from Ukwa and Izombe were significantly lower (*P* < 0.05) than the control. GST activity of *Piper guineense* from Ukwa and Izombe was higher compared with control *Gongronema latifolium* collected at Ukwa had higher GST activity than that of Izombe samples and control. The GST activity of *Pterocarpus mildbraedii* from Izombe recorded a significant higher activity (*P* < 0.05) than Ukwa and control. Fig. 9 shows the effect of gas flaring on plant glutathione peroxidase (GPx) activity. Glutathione peroxidase activity of *G. africanum* and *P. guineense* from Ukwa showed a significant (*P* < 0.05) higher activity when compared with control. The GPx of *G. latifolium* from Ukwa and Izombe recorded lower activities (*P* < 0.05) compared to control. The results of gas flaring effects on lactate dehydrogenase activity from plant leaves of test sites (Ukwa and Izombe), as well as the control site (Olokoro) are presented in Fig. 10. Activity of LDH of *G. africanum* from Ukwa and Izombe decreased significantly (*P* < 0.05) compared with the control. LDH of *P. guineense* and *G. latifolium* from Ukwa also decreased whereas that of *P. mildbraedii* from Ukwa and Izombe was higher than the control.

## DISCUSSION

The current study investigated the potential effect of gas flaring on soil and plant antioxidant enzymes activities. Enzymes may be useful as early indicators in the assessment of ecological risks due to environmental stress. This is because they immediately respond to alterations in biological functions in cells (Kaczynska *et al*. 2015). From the present study, soil sample from Ukwa exhibited significant higher increase (*P* < 0.05) in urease activity than Izombe and the control soil. This result may be due to the stabilisation capacity of the urease enzyme on polluted soil (Adetunji *et al*. 2017; Wyszkowska & Wyszkowski 2010). This stability makes the urease enzyme more resistant to environmental stress. Urease is an important extracellular enzyme that plays key role in nitrogen cycling in soil. Urease is also involved in mineralisation of organic nitrogen in the soil to ammonium that is easily accessible to plants which is considered as an important attribute of soil quality. This result agrees with previous reports (Adetunji *et al*. 2017; Wyszkowska & Wyszkowski 2010); Garcia-Gil *et al*. 2013) which reported increased activity of soil urease in response to different soil pollutants.

The activity of dehydrogenase enzyme (DHA) in soil is often used in evaluation of soil quality and the extent of soil contamination (Kaczynska *et al*. 2015; Curylo & Telesinski 2020). Izombe soil exhibited very large and significant (*P* < 0.05) dehydrogenase activities than Ukwa soil and the control soil sample from Olokoro which may signify some level stress from pollutants or redox state of the soil. Soil DHA plays a very important and significant role in oxidation of organic matter in the soil through the transformation of hydrogen from organic substrates to inorganic acceptors (Zhang *et al*. 2010; Curylo & Telesinski 2020). By doing so, DHA function as an important bio-indicator of microbiological redox system and therefore could be regarded as a good measure of the oxidative activities of microorganisms in the soil (Gu *et al*. 2009). This observation aligns with the reports of Wyszkowska and Wyszkowski (2010) which showed that the increased activity of DHA seen in hydrocarbon polluted soil may be due to the intensified proliferation of microorganisms and their activity in the soil. Plants require phosphorus for growth which they acquire from the soil. The phosphatase enzyme refers to a broad group of enzymes which hydrolyse organic phosphorous through the cleavage of phosphate bonds to available form of inorganic phosphate in soil (Lemanowicz *et al*. 2016). The enzyme is measured as both acid and alkaline phosphatase based on the differences in soil pH (Wyszkowska & Wyszkowski 2010; Adetunji *et al*. 2017). Acid phosphatase activity is predominantly found in acidic soil of pH less than 6, while alkaline phosphatase is found in neutral or alkaline soils of pH 6 to11. In the present report, phosphatase activity increased significantly in soil samples from Izombe soil compared to Ukwa site and the control. This could be due to the antioxidant enzyme activation in response to the pollutant-induced oxidative stress in soil from the gas flaring site. The obtained results is in line with previous reports (Wyszkowska & Wyszkowski 2010; Telesinski *et al*. 2018, Garciagil *et al*. 2013).

Any alteration in soil enzyme function is directly related to depletion of nutrient availability in the soil which may affect plant productivity indirectly or indirectly. The stimulation of enzymatic reactions in the soil in response to the pollutants in this case does not mean any improvement in soil fertility, as the pollutants from flared gas come with destructive influence on the soil structure as well as soil air, water and chemical properties of the soil (Ajugwo 2013; Garcia-Gil *et al*. 2013) which affects plant growth, yield and nutrients composition. Therefore, the most important and direct test for assessing the level of soil contamination is the plant antioxidant assay (Kavitha & Shailaja 2016) which provides more evidence on extend of damage caused by environmental stressors. For this reason, the plant antioxidant system is of special importance as bio-indicators to assess level of tolerance of plants to environmental pollutants. From the present study, SOD activity increased significantly in all plant vegetables studied. This is in agreement with the report of Ahmad *et al*. (2010) and Choudhury *et al*. (2013) who independently showed SOD activities in plants continued to increase with continued stress conditions by decreasing the ROS generated from the stress.

According to Ahmad *et al*. (2010), the ROS generated under stress conditions in plants is responsible for enhancing the activity of SOD enzymes which is in line with our findings. The SOD enzyme is seen as the first line of defense during oxidative stress in plant (Jain *et al*. 2020). The SOD enzyme is responsible for transformation of superoxide radicals that emerge as a result of stress in the plant into hydrogen peroxide. The production of hydrogen peroxide then triggers the increased synthesis of peroxidase and catalase enzyme activity (Sharma *et al*. 2012) to clear off the excessive hydrogen peroxide generated which then reduces the activity of SOD (Kusvuran *et al*. 2016). This may partially explain the observed reduction of SOD activity observed in *G. latifolium* and *P mildbraedii* plants leaves at Izombe gas flaring site. The activity of catalase enzyme was observed to increase significantly in all plants from Izombe site than in Ukwa and control site. The generation of hydrogen peroxide (H_2_O_2_) in the plants due to stress might be responsible for the stimulation of catalase as seen in Izombe site, however, a decrease in catalase activity observed at Ukwa gas flaring site could be due to the excessive accumulation of H_2_O_2_ to a toxic level that causes the degradation of the catalase enzyme by induced peroxisomal proteases (Lingard *et al*. 2009).

The present report showed an elevated activity (*P* < 0.05) of GST in plants from Ukwa than Izombe and control. GST enzymes are responsible for protecting plants from endogenous and exogenous toxic compounds. The enzyme has been linked with the detoxification of xenobiotics (foreign bodies) thereby limiting oxidative damage and other responses to stress in plants (Sharma *et al*. 2012; Dixon *et al*. 2010). The defensive activity of GST in plant is attained by covalently linking glutathione (GSH) to xenobiotic to form a GSH conjugate that is less toxic than the parent xenobiotic compound. The increased activity of GST in this report could be due to the presence of toxic pollutants or xenobiotic in the plant causing the increased synthesis of the GST enzymes in response to the effect. This agrees with the report of Dubey *et al*. (2010) suggesting that the expression of GST in plants is induced by various environmental stressors such as biotic and abiotic pollutants and heavy metals.

LDH is another important enzyme that plays a role in the detoxification of accumulated lactate in plants during stress condition. Plants grown at Izombe gas flaring site recorded higher activity (*P* < 0.05) of LDH than those grown at Ukwa when compared with the control. The gyoxalase pathway which is responsible for methylglyoxal transformation is the major source of accumulation of lactate in the plant to a toxic level (Jain *et al*. 2020). Methylglyoxal is a cytotoxic by-product produced from in plant during various biological reactions. The same is usually accumulated when plant is subjected to stress conditions especially from environmental pollutants (Yadav *et al*. 2005; Jain *et al*. 2020). Accumulation of methylglyoxal in plant cell disrupts the cellular homeostasis of plant by reacting with major macromolecules of the cell (Jain *et al*. 2020). In an attempt to detoxify methylglyoxal, the glyoxalase system (I and II) converts methylglyoxal directly to lactate which ultimately leads to a proportional accumulation of lactate in the system that is harmful to plants (Kowlgi & Chhabra 2015). Then, LDH plays a role in detoxification of the accumulated lactate. This shows that the lactate dehydrogenase enzyme might have some role in the mitigation of abiotic stress in plan exposed to environmental pollution. Hence, the increased in activity of LDH seen in our study could be due to overexpression of the enzyme as a way of increasing tolerance to lactate (Welchen *et al*. 2016) which aligns with previous reports suggesting that extent of oxidative damage in plants exposed to environmental stress is controlled by their antioxidant enzyme capacity (Ahmad *et al*. 2010; Mittler 2002). In other words, a constant balance is maintained between the antioxidant systems in plants and the ROS content in order to keep these radicals at levels compatible with cellular metabolism. The ability to maintain such balance determines the fate of plants when exposed to environmental pollutants which can affect its growth and productivity if altered.

## CONCLUSION

The soil enzymes and plant antioxidant system variability in this study is an indication that soil ecosystem and plants grown at these gas flaring sites are at risk due to long term stress load of the pollutants from the gas sites. It is possible that the excessive stimulation of the activity of soil enzymes and alterations of plant antioxidant defense mechanisms could be another means by which pollutants from the gas flaring are impacting on soil quality and crop yields in these communities. Therefore assessing the activity of soil and antioxidant enzymes could be employed as useful bio-indicators of soil degradation and monitorial indices for bioremediation.
